# Rurality, socioeconomic status, and psychosocial health outcomes during pregnancy

**DOI:** 10.1186/s12884-025-08492-1

**Published:** 2025-12-01

**Authors:** Katrina L. Wilhite, Jacob Gallagher, Alex Crisp, Jaemyung Kim, Andrea C. Kozai, Treah Haggerty, Kara M. Whitaker, Bethany Barone Gibbs

**Affiliations:** 1 West Virginia University, Morgantown, WV USA; 2 Iowa State University, Ames, IA USA; 3 University of Iowa, Iowa City, IA USA; 4 University of Pittsburgh, Pittsburgh, PA USA

## Abstract

**Background:**

Depressive symptoms, quality of life related to nausea/vomiting, and perceived stress (i.e., psychosocial health outcomes) tend to worsen in pregnancy. Yet whether these differ between rural and urban areas or across socioeconomic statuses during pregnancy remains unclear. We investigated whether there are differences in psychosocial health outcomes during pregnancy by rurality, socioeconomic status, and their intersectionality.

**Methods:**

Data were from Pregnancy 24/7, a pregnancy cohort study conducted from 2020 to 2024 among 497 participants recruited from three sites in the United States (Iowa City, Iowa; Pittsburgh, Pennsylvania; and Morgantown, West Virginia). Participants attended study visits in each trimester where they self-reported depressive symptoms (Center for Epidemiology Studies Depression), nausea- and vomiting-related (NV) quality of life (Nausea and Vomiting of Pregnancy Quality of Life Questionnaire), and perceived stress (Perceived Stress Scale). Rurality was based on home address as urban, micropolitan, and small town rural. Socioeconomic status was measured at the neighborhood level via the national Area Deprivation Index and at the individual level via latent classes (i.e., high, middle, or low) constructed from education, income, and insurance status. Unadjusted and adjusted mixed-effects linear regression models separately examined differences in psychosocial health outcomes between rurality and socioeconomic status. Intersectionality, defined as a compounding effect, was explored by evaluating an interaction term between rurality and socioeconomic status.

**Results:**

Though participants who were rural, had greater Area Deprivation Index, and had lower individual-level socioeconomic status had higher (worse) psychosocial health outcome scores in unadjusted models, significant differences only persisted across individual-level socioeconomic statuses after adjustment. Scores for high vs. low socioeconomic status were 5.41 vs. 7.56 for depressive symptoms (*p* < 0.001). Scores for high vs. middle socioeconomic status were 80.29 vs. 87.84 for NV quality of life (*p* = 0.02) and 12.15 vs. 14.16 for stress (*p* < 0.001). When exploring intersectionality, individuals in rural areas and the lowest socioeconomic latent class experienced the poorest psychosocial health outcomes; however, non-significant interaction terms suggested intersectionality was not present.

**Conclusion:**

Individuals with low individual-level socioeconomic status had poorer psychosocial health in pregnancy. Population-specific strategies to improve psychosocial health in low socioeconomic status pregnant people should be explored, including in rural settings.

**Supplementary Information:**

The online version contains supplementary material available at 10.1186/s12884-025-08492-1.

## Introduction

Pregnant individuals experience a series of physical, social, and hormonal changes that can negatively influence psychosocial health outcomes, including depression, quality of life, and stress. In the United States, depression during pregnancy affects between 10 and 20% of individuals, which, if not addressed, can continue postpartum [1]. Health-related quality of life is typically lower for pregnant individuals as compared to the general population, especially as related to nausea and vomiting [2, 3]. Additionally, experiencing mild to moderate stress is reported by many pregnant individuals [4, 5]. Poor psychosocial health is related to adverse maternal and infant outcomes such as hypertensive disorders of pregnancy, preterm delivery, and lower breastfeeding initiation [1, 6]. Further, if poor psychosocial health outcomes remain untreated postpartum, there is an increased risk of maternal suicide, poor mother-child bonding, and behavioral, cognitive, and health problems in the child [1, 4, 7]. These maternal-child consequences highlight the need to identify risk factors for poor psychosocial health in pregnancy to inform interventions to improve maternal-child outcomes.

Whether psychosocial health outcomes during pregnancy differ across rurality is unclear due to limited research, though such differences could be contributing to the poorer maternal and child outcomes experienced by pregnant individuals living in rural areas compared to their urban counterparts [8]. One relevant analysis using data from the Pregnancy Risk Assessment Monitoring System (PRAMS) surveillance study, and including 17,229 participants from 14 states across the United States, found that individuals from rural areas were 21% more to likely to report depression in their recent pregnancy or postpartum compared to those from urban areas [9]. Another study, from Indonesia and among 400 rural and 400 urban pregnant participants recruited from antenatal care clinics, found that quality of life related to physical health, mental health, and social and environmental factors were similar between those living in rural versus urban areas [10]. Yet, these studies measured psychosocial health outcomes retrospectively or at only one time point during pregnancy and country-specific differences may influence the results. Few studies have described differences in experiencing stress during pregnancy in rural versus urban areas, but pregnant rural individuals have reported transportation difficulties, isolation, unemployment, financial stress, and distance to birthing centers as stressors [11–13]. Taken together, the limited available research evaluating differences in psychosocial health outcomes during pregnancy by rurality warrants further investigation.

Socioeconomic status (SES) is another risk factor that could be contributing to poor psychosocial health outcomes during pregnancy. For example, a handful of studies from the United States (specifically California; *n* = 198), northern Germany (*n* = 792), and the Netherlands (*n* = 5,398) have found that those with lower SES as defined by education, income, marital status, home ownership, and employment more often experience depression during pregnancy than those with higher SES [14–16]. Similarly, a systematic review examining factors that influence quality of life in pregnant individuals found that those without social or economic hardship experienced a better quality of life than those who reported having social or economic problems [2]. Stress during pregnancy by SES is less studied. In the general population, certain types of stress including perceived stress tend to be higher in those with lower SES [17]. Additionally, most study designs examining differences in psychosocial health outcomes across SES groups are cross-sectional and do not assess these outcomes across gestation. As both individual-level and neighborhood-level socioeconomic status are associated with poorer maternal-fetal outcomes in the United States [18, 19], differences in psychosocial health outcomes by SES at both of these levels across pregnancy need to be explored.

Furthermore, the potential intersectionality of rurality and SES with psychosocial health outcomes should be evaluated. Approximately 15.9% of rural individuals in the United States live in poverty compared to 11.9% of individuals living in urban areas [20]. As evidenced by studies reporting on stressors in rural pregnant individuals, some of the key stressors include socioeconomic hardship, such as financial troubles and lack of employment [11]. Living in both a rural area and having low SES may have compounding effects on psychosocial health outcomes, though research on whether this intersectionality results in poorer psychosocial health outcomes during pregnancy is lacking.

To address these research gaps, the current investigation sought to evaluate potential rural-urban and SES differences in psychosocial health outcomes (i.e., depressive symptoms, quality of life, and stress) in a pregnancy cohort assessed across trimesters. Additionally, we aimed to understand the intersectionality between rurality and SES on psychosocial health outcomes during pregnancy. We hypothesized that individuals from rural areas or those with lower SES would report poorer psychosocial health outcomes than their counterparts in urban areas or those with higher SES, respectively. We further hypothesized that individuals living in rural areas with low SES would experience poorer psychosocial health outcomes compared to those residing in more urban areas, having higher SES, or both.

## Methods

### Participants

This secondary analysis used data from the Pregnancy 24/7 cohort study [21]. Pregnancy 24/7 was a longitudinal study of 500 participants conducted at three study sites (University of Iowa, West Virginia University, University of Pittsburgh) between February 2021 and May 2024. The study aimed to examine the associations of sedentary behavior, sleep, and 24-hour activity (composition of physical activity, sedentary behavior, and sleep) with adverse pregnancy outcomes. Eligibility criteria included being 18–45 years old, less than 13 weeks gestation, not using blood pressure- or glucose-lowering medications, not being treated for a sleep disorder, no severe limitations to mobility, and not being treated for any serious medical conditions that would markedly alter 24-hour activity (e.g., severe heart or lung disease). Participants were asked to attend three study visits, one during each trimester of pregnancy (gestational ages of 10^0^−12^6^ weeks, 20^0^−22^6^ weeks, and 32^0^−34^6^ weeks). At the first visit, participants were asked to attend an in-person clinical assessment where height and weight were measured. Participants with barriers to attending the in-person visit (such as lack of transportation) were allowed to complete the first visit remotely through a modified protocol to increase enrollment of harder-to-reach populations. Assessment visits during the second and third trimester were completed remotely for all participants. For all three visits, participants completed questionnaires related to psychosocial health outcomes. This study was conducted in accordance with the principles of the Belmont Report.

### Rurality

Rurality was defined as urban (1–3), micropolitan rural (4–6), and small town rural (7–10) based on participants’ Rural-Urban Commuting Area (RUCA) code [22]. RUCA codes determine rurality based on census tract blocks, considering population, urbanization, and commuting patterns. Participants’ addresses were entered into the geocoding tool developed by the United States Department of Agriculture (https://geomap.ffiec.gov/ffiecgeomap/) and found in the 2010 RUCA database.

### Socioeconomic status

SES was measured at the neighborhood and individual levels. Neighborhood level SES was measured by the national Area Deprivation Index percentiles using the Neighborhood Atlas tool designed by the US Census Bureau using participants’ addresses reported during the study [23, 24]. Neighborhoods are measured at the census block level and include theoretical information regarding income, education, employment, and housing quality. Higher percentiles indicate more neighborhood disadvantage. Participants were separated into sample-specific tertiles.

Individual level SES was determined by latent class analysis using the poLCA package, version 1.6.0.1, in R, which can account for missing data [25]. Participants’ self-reported insurance status (Private or Medicaid/Medicare), income (<$49,999; $50,000 - $99,000; $100,000-$149,999; ≥ $150,000), and education (pre-baccalaureate, baccalaureate, post-baccalaureate) at study visits were used. The number of classes was determined by Bayesian Information Criteria (BIC) and entropy best-fit model statistics.

Fit-criteria statistics identified a 3-class model as being the most appropriate due to having the lowest BIC values (2698.28) and an acceptable entropy of 0.76. The first class (High SES), 34.5% of the sample, was characterized by most participants having a post-baccalaureate degree, being in the highest income quartile, and all participants were covered by Private health insurance. The second class (Middle SES), 47.3% of the sample, was characterized by approximately half of the participants having a baccalaureate degree and being in the second income quartile. Additionally, almost all participants were covered by Private health insurance. The third class (Low SES), 18.2% of the sample, was characterized by most participants having a pre-baccalaureate education, being in the lowest income quartile, and receiving Medicaid/Medicare for insurance. Details regarding fit-criteria statistics and latent class characteristics can be found in Additional file1.

### Psychosocial health outcomes

Psychosocial health outcomes measured during Pregnancy 24/7 included depressive symptoms, nausea- and vomiting-related (NV) quality of life, and stress. Depressive symptoms were measured using the Center for Epidemiology Studies Depression 10-item Scale (CES-D) [26], which ranges from 0 to 30 with higher scores indicating higher depression risk. NV quality of life was measured using the Nausea and Vomiting of Pregnancy Quality of Life Questionnaire (NVPQoL) [27, 28], which ranges from 30–210 with higher scores indicating a worsening NV quality of life. Stress was measured using the Perceived Stress Scale 10-item version (PSS) [29], which ranges from 0–40 with higher values indicating higher perceived stress. All questionnaires have been found valid and reliable in pregnant populations [28, 30–33]. Participants were included for each psychosocial health outcome if they had valid data from at least one time point. A questionnaire was considered valid if it had no missing questions.

### Covariates

Covariates included self-reported measures of age, marital status (Married/Living in a Marriage-like Relationship or Single/Divorce/Widowed), the social constructs of race (Asian, Black/African American, Multiracial, Native Hawaiian or other Pacific Islander, or White) and ethnicity (identified as Hispanic/Latino), and number of children living at home (none, 1, 2+). Pre-pregnancy body mass index (BMI, kg/m^2^) was determined using measured height from the in-person visit and pre-pregnancy weight abstracted from medical records. Height was the average of two measures without shoes using a stadiometer. If the height difference was > 0.5 cm a third measurement was taken, and the two closest measurements were averaged. In instances where participants opted for the remote clinical assessment (*n* = 75), height and weight were self-reported.

### Statistical analysis

Analyses were conducted using Stata v17.0 (College Station, TX, United States) and R software, version 4.4.1 (R Foundation for Statistical Computing, Vienna, Austria). First, unadjusted mixed-effects linear regression models described changes in psychosocial health outcome scores by trimesters. Then, unadjusted mixed-effects linear regression models using the lme4 package, version 1.1.35.5 [34], were used to examine whether there were differences in psychosocial health outcomes across rural and socioeconomic categories (i.e., Area Deprivation Index tertiles or individual-level latent classes), with a random intercept to account for repeated measures within participants across trimesters of pregnancy and a fixed effect for trimester. Additionally, the intersectionality (defined as a compounding effect) of rurality and SES on psychosocial health outcomes in pregnant individuals was evaluated by interaction terms between rurality and SES. Then, models were adjusted for age, pre-pregnancy BMI, marital status, race, site, and number of children living at home. Unadjusted models are presented for all outcomes, as our objective is to understand whether there were psychosocial health differences across rurality and SES to inform public health needs. In this way, we provide findings that estimate the total observed differences rather than controlling for covariates that contribute to outcomes experienced in different rural and socioeconomic settings. Adjusted analyses are also presented to understand the contribution of potential confounders to differences. Finally, we considered excluding the Pittsburgh site from our analyses due to the limited number of participants residing in a rural area (*n* = 3). Because the Pittsburgh area has a wide range of socioeconomic diversity, we decided to include all three sites in the analyses as our primary approach and to present results without the Pittsburgh participants as a sensitivity analysis.

## Results

### Participant characteristics and psychosocial health outcomes by trimester

There were 497 participants included in this study. A total of 497 participants had at least one complete CES-D and NVPQoL questionnaire, and 496 participants had at least one complete PSS questionnaire. A summary of participant characteristics is reported in Table 1. Differences were observed across rurality groups (urban, micropolitan rural, and small town rural) for most baseline characteristics (*P <* 0.05). Micropolitan rural participants tended to be younger, were more likely to be single, divorced or widowed, have Medicaid/Medicare insurance, and be from the West Virginia study site compared to urban and small town rural groups. On the other hand, urban participants were more likely to have no children in the home, have higher income, greater education, and live in the least disadvantaged neighborhoods compared to the micropolitan and small town rural groups. Retention and valid survey completion were excellent (Table 2), with psychosocial health outcome data available in 99% of participants from the first trimester visit, 90% of participants from the second trimester visit, and 88% from the third trimester visit. Average depressive symptoms and perceived stress scores were similar across pregnancy (within one point), though they were statistically higher in the first vs. second trimesters. NV quality of life scores varied more. They were least favorable in the first trimester and improved in the second and third trimesters, which is coherent with decreased nausea after the first trimester.

**Table 1:** Summary of participant characteristics

	Overall (*N* = 497)	Urban (*N* = 385)	Micropolitan Rural (*N* = 43)	Small Town Rural (*N* = 69)	*P*
Age (years)	30.6 (4.53)	31.0 (4.35)	27.8 (5.10)	30.7 (4.65)	< 0.001
Race					0.05
Asian	25 (5.0%)	24 (6.2%)	1 (2.3%)	0 (0%)	
Black/African American	28 (5.6%)	27 (7.0%)	0 (0%)	1 (1.4%)	
Multiracial	15 (3.0%)	14 (3.6%)	0 (0%)	1 (1.4%)	
Native Hawaiian or other Pacific Islander	1 (0.2%)	1 (0.3%)	0 (0%)	0 (0%)	
White	428 (86.3%)	319 (83.1%)	42 (97.7%)	67 (97.1%)	
Hispanic Ethnicity					0.36
Yes	21 (4.2%)	15 (3.9%)	1 (2.3%)	5 (7.2%)	
No	476 (95.8%)	370 (96.1%)	42 (97.7%)	64 (92.8%)	
Marital Status					0.01
Married/Marriage-like	447 (89.9%)	350 (90.9%)	33 (76.7%)	64 (92.8%)	
Single/Divorced/Widowed	50 (10.1%)	35 (9.1%)	10 (23.3%)	5 (7.2%)	
Children in the Home					< 0.001
None	197 (39.7%)	166 (43.2%)	15 (34.9%)	16 (23.2%)	
1	174 (35.0%)	136 (35.3%)	14 (32.6%)	24 (34.8%)	
2+	116 (23.4%)	75 (19.5%)	12 (27.9%)	29 (42.0%)	
Pre-Pregnancy BMI (kg/m ^2^)	27.4 (6.75)	27.0 (6.48)	27.7 (7.45)	29.3 (7.50)	0.03
Income					0.01
First Quartile (Lowest Income)	85 (17.1%)	57 (14.8%)	11 (25.6%)	17 (24.6%)	
Second Quartile	140 (28.2%)	101 (26.3%)	15 (34.9%)	24 (34.8%)	
Third Quartile	137 (27.6%)	113 (29.4%)	8 (18.6%)	16 (23.2%)	
Fourth Quartile (Highest Income)	121 (24.4%)	106 (27.6%)	6 (14.0%)	9 (13.0%)	
Missing	14 (2.8%)	8 (2.1%)	3 (7.0%)	3 (4.3%)	
Education					< 0.001
Pre-baccalaureate	121 (24.4%)	72 (18.8%)	20 (46.5%)	29 (42.0%)	
Baccalaureate	166 (33.5%)	125 (32.6%)	11 (25.6%)	30 (43.5%)	
Post-baccalaureate	210 (42.3%)	188 (48.8%)	12 (27.9%)	10 (14.5%)	
Insurance Status					< 0.01
Medicaid/Medicare	81 (16.3%)	50 (13.0%)	15 (34.9%)	16 (23.2%)	
Private	415 (83.5%)	334 (86.8%)	28 (65.1%)	53 (76.8%)	
Missing	1 (0.2%)	1 (0.3%)	0 (0%)	0 (0%)	
Area Deprivation Index ^a^					< 0.001
Most Disadvantage	174 (35.1%)	96 (25.0%)	32 (74.4%)	46 (66.7%)	
Middle Disadvantage	166 (33.4%)	141 (36.6%)	7 (16.3%)	18 (26.1%)	
Least Disadvantage	147 (29.6%)	139 (36.2%)	4 (9.3%)	4 (5.8%)	
Missing	10 (2.0%)	9 (2.3%)	0 (0%)	1 (1.4%)	
Study Site					< 0.001
Iowa	250 (50.4%)	188 (49.0%)	13 (30.2%)	49 (71.0%)	
Pittsburgh	125 (25.2%)	122 (31.7%)	2 (4.7%)	1 (1.4%)	
West Virginia	122 (24.6%)	75 (19.5%)	28 (65.1%)	19 (27.5%)	

^a^Data was split into sample-specific tertiles. Values presented as mean (standard deviation) or frequency (%). Income quartiles (<$49,999; $50,000 - $99,000; $100,000-$149,999; >= $150,000

**Table 2:** Average scores for psychosocial outcomes across trimesters of pregnancy

	CES-D 10			NVPQoL			PSS			
Trimester	*n*	Score	*P*	*n*	Score	*P*	*n*	Score	*P*	
1	495	6.92 (0.21)	Reference	494	104.83 (1.47)	Reference	494	14.85 (0.30)	Reference	
2	452	6.44 (0.21)	0.008	451	77.11 (1.52)	<0.001	451	14.26 (0.31)	0.018	
3	443	7.23 (0.21)	0.094	442	85.92 (1.53)	<0.001	442	14.54 (0.31)	0.223	

### Differences in psychosocial health outcomes by rurality and socioeconomic categories

As shown in Table 3, participants living in small town rural areas reported more depressive symptoms compared to their urban counterparts (7.87 vs. 6.59 points, *p* = 0.04). For the Area Deprivation Index, participants living in the most disadvantaged areas reported higher depressive symptoms (8.00 vs. 6.17 points, *p* < 0.001), poorer NV quality of life (96.52 vs. 85.94 points, *p* < 0.001), and higher perceived stress (15.82 vs. 13.76 points, *p* = 0.01) than those living in the least disadvantaged areas. When examining individual-level SES measured by latent classes, those in the Middle and Low SES classes reported higher depressive symptoms, poorer NV quality of life, and higher perceived stress than those in the High SES class (all *p* < 0.001, see Table 3).

**Table 3:** Unadjusted psychosocial health outcomes by rurality and socioeconomic status across pregnancy

Group	CES-D 10 Score	*P*	NVPQoL Score	*P*	PSS Score	*P*
Rurality						
Urban (*n* = 385)^a^	6.59 (0.21)	Reference	87.59 (1.45)	Reference	14.21 (0.31)	Reference
Micropolitan Rural (*n* = 43)	7.77 (0.62)	0.17	93.99 (4.45)	0.36	15.93 (0.92)	0.18
Small Town Rural (*n* = 69)	7.87 (0.49)	0.04	96.00 (3.44)	0.06	15.59 (0.72)	0.18
Area Deprivation Index						
Least Disadvantage (*n* = 147)	6.17 (0.33)	Reference	85.94 (2.32)	Reference	13.76 (0.49)	Reference
Middle Disadvantage (*n* = 166)^a^	6.33 (0.31)	0.93	84.80 (2.19)	0.93	13.91 (0.46)	0.97
Most Disadvantage (*n* = 174)	8.00 (0.31)	< 0.001	96.52 (2.17)	< 0.001	15.82 (0.46)	0.01
Individual Socioeconomic Status Latent Class						
High SES (*n* = 172)^a^	5.56 (0.29)	Reference	82.26 (2.05)	Reference	12.83 (0.43)	Reference
Middle SES (*n* = 235)	7.25 (0.26)	< 0.001	92.28 (1.88)	< 0.001	15.30 (0.39)	< 0.001
Low SES (*n* = 90)	8.70 (0.42)	< 0.001	97.03 (3.07)	< 0.001	16.31 (0.63)	< 0.001

*CES-D 10* Center for Epidemiologic Studies Depression 10-item Scale (range: 0–30). Higher scores indicate a higher risk of depression. *NVPQoL* Nausea and Vomiting of Pregnancy Quality of Life (range 30–210). Higher scores indicate worsening of NV quality of life. *PSS* Perceived Stress Scale 10-item version (range: 0–40). Higher scores indicate higher perceived stress. All values are expressed as mean estimates (standard error). Pairwise comparisons were conducted via Tukey’s post-hoc test. Latent classes consist of individual-level socioeconomic factors (i.e., income, education, insurance status)

^a^Indicates one less participant included in the PSS score

After adjusting for age, pre-pregnancy BMI, marital status, race, site, and number of children living at home, there were no differences in psychosocial health outcomes by rurality or Area Deprivation Index (Table 4). However, poorer scores for those in the Middle SES class remained statistically significant for depressive symptoms, NV quality of life, and perceived stress compared to those in the High SES class (all *p* < 0.05). Those in the Low SES class had more depressive symptoms (*p* < 0.001) compared to those in the High SES class, but differences between the two classes in NV quality of life and perceived stress were attenuated after adjustment.

**Table 4:** Adjusted psychosocial health outcomes by rurality and socioeconomic status across pregnancy

Group	CES-D Score	*P*	NVPQoL Score	*P*	PSS Score	*P*
Rurality						
Urban (*n* = 385)*	6.99 (0.88)	Reference	88.34 (6.24)	Reference	13.84 (1.32)	Reference
Micropolitan Rural (*n* = 43)	7.51 (1.07)	0.72	91.09 (7.64)	0.84	14.80 (1.60)	0.61
Small Town Rural (*n* = 69)	8.15 (1.00)	0.08	95.37 (7.15)	0.15	15.04 (1.51)	0.29
Area Deprivation Index						
Least Disadvantage (*n* = 147)	6.77 (0.94)	Reference	88.07 (6.65)	Reference	13.45 (1.42)	Reference
Middle Disadvantage (*n* = 166)*	6.74 (0.91)	1.00	85.78 (6.46)	0.75	13.55 (1.38)	0.99
Most Disadvantage (*n* = 174)	7.64 (0.91)	0.18	93.85 (6.45)	0.21	14.55 (1.37)	0.29
Individual Socioeconomic Status Latent Class						
High SES (*n* = 172)*	5.41 (0.96)	Reference	80.29 (6.88)	Reference	12.15 (1.45)	Reference
Middle SES (*n* = 235)	6.91 (0.91)	< 0.001	87.84 (6.57)	0.02	14.16 (1.38)	< 0.001
Low SES (*n* = 90)	7.56 (0.92)	< 0.001	91.46 (6.58)	0.05	14.12 (1.38)	0.12

*CES-D 10* Center for Epidemiologic Studies Depression 10-item Scale (range: 0–30). Higher scores indicate a higher risk of depression. *NVPQoL* Nausea and Vomiting of Pregnancy Quality of Life (range 30–210). Higher scores indicate worsening of NV quality of life. *PSS* Perceived Stress Scale 10-item version (range: 0–40). Higher scores indicate higher perceived stress. Adjusted for age, pre-pregnancy body mass index, marital status, race, and number of children living at home. All values are expressed as mean estimates (standard error). Pairwise comparisons were conducted via Tukey’s post-hoc test. Latent classes consist of individual-level socioeconomic factors (i.e., income, education, insurance status)

^a^Indicates one less participant included in the PSS score

### Differences in psychosocial health outcomes and the intersectionality of rurality and socioeconomic categories

**Fig. 1:**
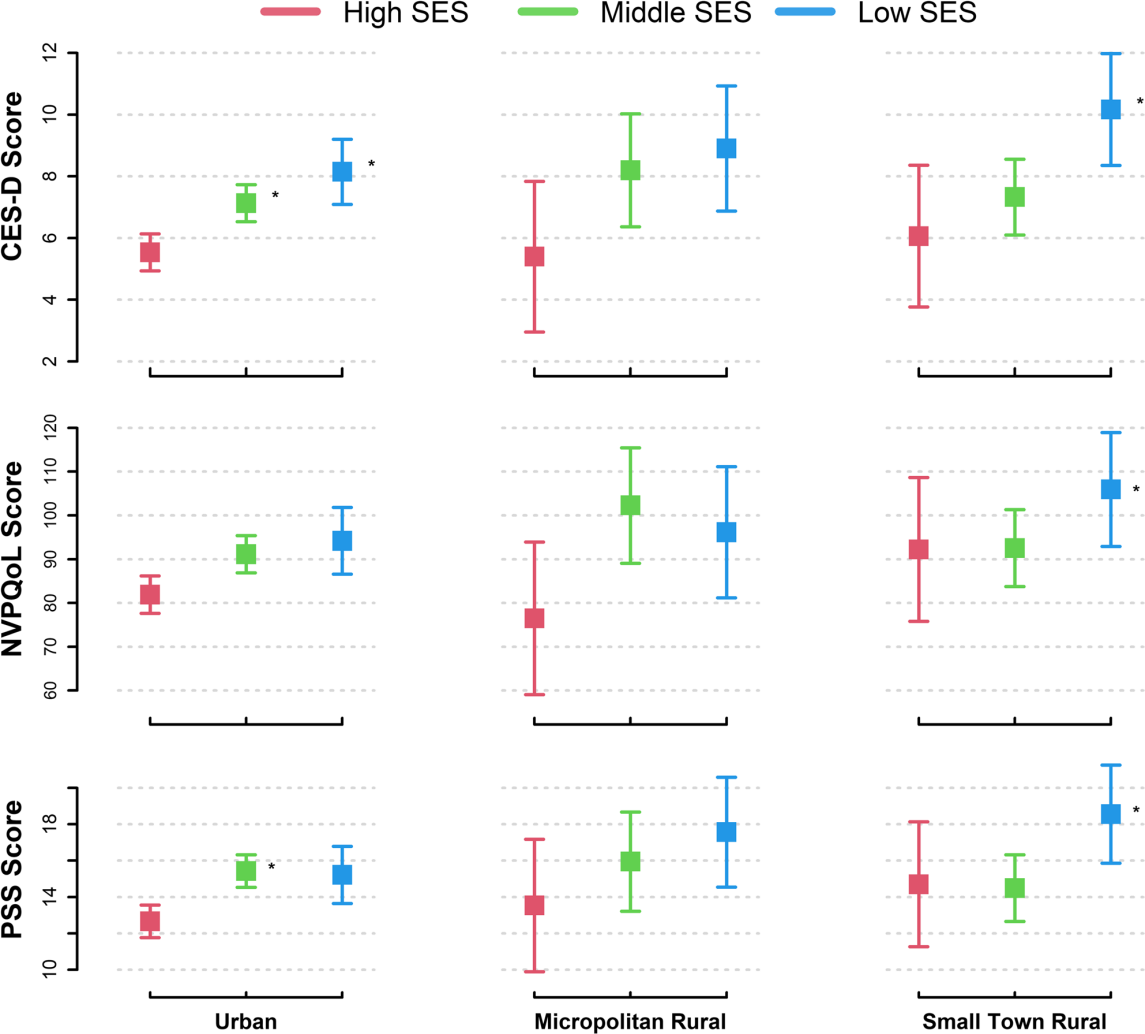
Unadjusted Differences in Psychosocial Health Outcomes Considering the Intersectionality of Rurality and Individual Socioeconomic Status across Pregnancy

**Fig. 2:**
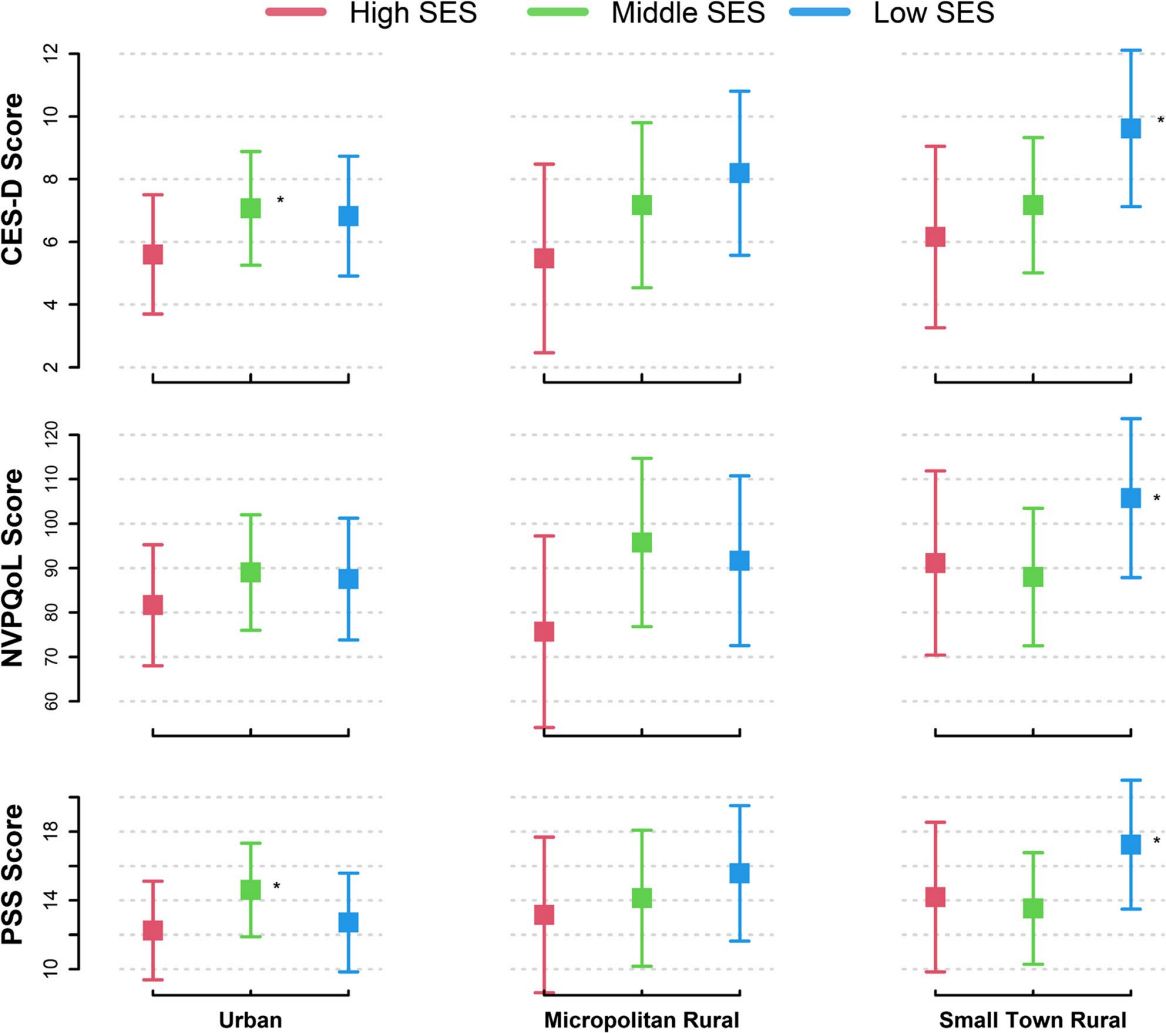
Adjusted Differences in Psychosocial Health Outcomes Considering the Intersectionality of Rurality and Individual Socioeconomic Status Across Pregnancy

Our results suggested no intersectionality as the overall interaction terms between rurality and individual SES latent classes were not significant in unadjusted (*p*-for-interaction: 0.60 for depressive symptoms; 0.32 for NV quality of life; and 0.20 for stress, Additional file 2) or adjusted analyses (p-for-interaction: 0.33 for depressive symptoms; 0.17 for NV quality of life; and 0.05 for stress, Additional file 3). However, it is important to note that, though there was not a compounding effect, the poorest scores for depressive symptoms, NV quality of life, and perceived stress were observed in participants living in small town rural areas with low SES (Fig. 1). These poorest psychosocial health outcomes persisted in adjusted models (Fig. 2). No differences were detected across intersectional groups defined by rurality and neighborhood SES (Additional files 2 and 3).

Sensitivity analyses, which excluded Pittsburgh participants (*n* = 125), showed no difference compared to the primary analyses (Additional file 4).

## Discussion

We found that greater rurality and lower SES were generally associated with higher depressive symptoms, poorer NV quality of life, and higher perceived stress in pregnant individuals. However, only differences across individual level SES and psychosocial health outcomes remained after adjustment, highlighting this as a potent risk factor. Further, though not meeting the definition of intersectionality, we observed that pregnant individuals with the combination of greater rurality and lower individual level SES had the poorest psychosocial health outcomes. This indicates that individuals with both factors may be most in need of support during pregnancy.

Psychological health is a growing public health concern in the United States, with pregnant individuals an especially vulnerable population [1–3]. For example, from 2020 nationwide surveillance data, the estimated prevalence of depression in adults was 18.5% and these rates were higher in women (23.4%), younger adults (21.5%), and in West Virginia (27.5%) which had the highest rate of any state; rates of depression in Pennsylvania (20.9%) and Iowa (18.1%) were closer to the national average [35]. Though comparable research to the current report investigating rural-urban differences in depression, NV quality of life, and perceived stress among pregnant women in the United States is limited, our finding of greater depressive symptoms scores in rural participants is consistent with the 21% increase in reported depression during the most recent pregnancy among women residing in rural compared to urban areas in PRAMs [9]. There are several plausible reasons why pregnant individuals living in more rural areas may experience poorer psychosocial health outcomes than their urban counterparts. Individuals living in rural settings often face unique stressors, including limited access to healthcare services, greater distances to healthcare facilities, social isolation, and transportation challenges [11, 12, 36]. Specifically for pregnant women, there is less perceived access to quality support groups to help with pregnancy and postpartum [37, 38]. Several lifestyle behaviors, such as physical activity, alcohol use, and tobacco use, tend to be worse in rural communities compared to urban ones. These behaviors act bidirectionally with stress and depression, making lifestyle interventions a potential strategy for comprehensive care that promotes psychosocial health in rural pregnant women [39–42]. Pregnant individuals living in rural areas also tend to use more recreational drugs than their urban counterparts [43]. Strategies to improve access to healthcare, increase availability and quality of social support networks, and promote healthy lifestyle behaviors may be helpful intervention elements to positively influence psychosocial health outcomes in rural populations.

Similarly, a variety of factors may contribute to our findings of worse psychosocial health in pregnant individuals with lower neighborhood SES, measured by the national Area Deprivation Index. The Area Deprivation Index is an extensively validated proxy measure for neighborhood-level SES that incorporates census block group measures of income, education, employment, and housing quality. Area Deprivation Index has been associated with certain health outcomes (such as cardiovascular disease, accelerated aging, and poor healthcare access) independently from individual-level SES [23, 44]. Our study offers new findings where lower neighborhood level SES by this measure was related to poorer psychological health outcomes during pregnancy in Iowa, Pennsylvania, and West Virginia in unadjusted models. Lower psychosocial health outcomes among those living in the most disadvantaged neighborhoods in these areas may be due to environmental and pregnancy-specific challenges. One possible explanation informed by social-disorganization theory is that neighborhoods with low SES are typically characterized by higher rates of delinquency, crime, and poverty, and exhibit less social cohesion [45, 46]. Enhancing neighborhood trust and cohesion or implementing interventions in specific areas outside of their immediate neighborhood may be possible strategies to address these differences.

Our finding that pregnant individuals with lower individual level SES, measured by latent class, have poorer psychosocial health outcomes than their higher SES peers aligns with previous research [16, 17, 20]. Poor psychosocial health outcomes, particularly depressive symptoms, during pregnancy are associated with financial and housing stressors, lack of familial social support, and negative life events [47]. Additionally, those with lower SES tend to engage in less desirable behaviors that can negatively affect psychosocial health outcomes, such as participating in less physical activity, having poorer sleep quality, and continuing to smoke during pregnancy [48–50]. Also, those with lower SES tend to receive care and be referred less often to treatment for mental health-related factors than those with higher SES [51]. Potential strategies to improve psychosocial health outcomes in individuals with low SES may include increasing awareness of programs (e.g., Women, Infants & Children Program) that can support them during and after pregnancy, providing access to strong social support groups, and encouraging healthy lifestyle behaviors. Also, our results found that poorer psychosocial health outcomes in lower individual level SES categories remained after adjustment, suggesting that this population is likely most in need of intervention. Programs and resources should prioritize support for psychosocial health in pregnant individuals with low individual SES.

When exploring intersectionality, the combination of greater rurality and lower individual SES consistently resulted in the poorest psychosocial health outcomes in pregnant individuals but did not compound the observed differences. The interpretation is that pregnant people with lower individual level SES have lower psychosocial health outcomes regardless of rurality status and vice versa (see Figures). Still, the interaction term testing for intersectionality approached significance in adjusted models for perceived stress (*p* = 0.05) and, moreover, the unfavorable differences of both add together to result in the worst psychosocial health among those with low individual level SES living in rural areas. Future research should repeat our analyses with a well-powered sample to confirm these results and investigate intervention strategies that address pregnant individuals who live in rural settings and have low individual level SES, as they may need the most psychological health support. Low-cost interventions implemented in local or remote settings to increase accessibility, promote healthy lifestyle behaviors, and incorporate a strong social support system may be especially appropriate.

Our study had several strengths. First, we presented a comprehensive understanding of psychosocial health outcomes during pregnancy by measuring multiple components of psychosocial health (i.e., depressive symptoms, NV quality of life, and perceived stress) prospectively and across pregnancy. Next, our sample was recruited from three different study sites, increasing participant variability and external validity. Finally, our SES variables were robust. We were able to investigate SES differences at the neighborhood level using the national Area Deprivation Index tool, as well as a multidimensional individual SES measure that included income, education, and insurance status. Our study examined rurality and SES individually, as well as the intersection of rurality and SES. This is important because it is difficult to disentangle whether rurality or low SES is the primary driver for poor psychosocial health outcomes during pregnancy. By conducting this series of analyses, it appears that low individual SES is likely the main driver of poor psychosocial health in pregnant populations, though rurality may contribute to additional differences. Limitations include that, as an observational research study, we cannot determine that differences in psychological health outcomes were caused by rurality and SES; we can only establish that the differences were detected. Next, our rural sample in this secondary analysis was small, and this limited our ability to detect significant differences, including interactions. Non-significant differences and interaction terms should be interpreted with this in mind. Further, generalizability of our findings is limited by the fact that participants had to meet eligibility criteria and enroll in the Pregnancy 24/7 observational cohort research study as well as by the characteristics of the sample that included greater than 75% with a college degree and 86.3% who self-identified as White. Finally, the self-report questionnaires for the psychosocial health outcomes may be subject to social desirability bias.

## Conclusion

This study suggests that those living in rural areas and with lower SES, both at a neighborhood and individual level, have higher depressive symptoms, poorer NV quality of life, and higher perceived stress than those living in urban areas and with higher SES. Poorer psychological health outcomes were most consistently observed among lower SES groups during pregnancy in adjusted models, and intersectionality between rurality and SES was not observed. Future research should investigate modifiable factors contributing to these psychosocial health differences, as well as strategies to improve psychosocial health outcomes in populations that are most in need. This will support the long-term goal to design and test interventions incorporating these population-specific strategies to reduce adverse psychosocial health outcomes in rural areas and those with low SES.

## Supplementary Information

Additional file 1. Latent class analysis best-fit criterion statistics and class characteristics.

Additional file 2. Unadjusted Differences in Psychosocial Outcomes by Rurality and Socioeconomic Categories Across Pregnancy.

Additional file 3. Adjusted Differences in Psychosocial Outcomes by Rurality and Socioeconomic Categories Across Pregnancy.

Additional file 4. Sensitivity Analyses (Excluding Pittsburgh site).

## Data Availability

The datasets used and/or analyzed during the current study are available from the corresponding author on reasonable request.

## Abbreviations

SES: socioeconomic status
RUCA: Rural-urban commuting area code
CES-D: Center for Epidemiologic Studies Depression Scale
NVPQoL: Nausea and Vomiting during Pregnancy Quality of Life
PSS: Perceived Stress Scale
BMI: body mass index
