# Supplementary material for: Rurality, socioeconomic status, and psychosocial health outcomes during pregnancy
Source: BMC Pregnancy Childbirth. 2025 Dec 1;26:23. doi: 10.1186/s12884-025-08492-1 (PMC12777491; doi:10.1186/s12884-025-08492-1)
Supplement: Supplementary file 1 — Additional file 1. Latent class analysis best-fit criterion statistics and class characteristics. [file 12884_2025_8492_MOESM1_ESM.docx]

**Additional File 1.** Latent class analysis best-fit criterion statistics and class characteristics.

| **Model** | **log-likelihood** | **resid. df** | **BIC** | **AIC** | **likelihood-ratio** | **Entropy** |
| --- | --- | --- | --- | --- | --- | --- |
| Model 1 | -1486.818 | 36 | 3023.306 | 2989.637 | 451.401486 | - |
| Model 2 | -1297.523 | 27 | 2700.592 | 2629.046 | 72.810372 | 0.869 |
| **Model 3** | -1268.428 | 18 | 2698.280 | 2588.857 | 14.621294 | 0.763 |
| Model 4 | -1263.988 | 9 | 2745.276 | 2597.975 | 5.739699 | 0.617 |
| Model 5 | -1261.786 | 0 | 2796.750 | 2611.572 | 1.336588 | 0.562 |
| Model 6 | -1261.312 | -9 | 2851.679 | 2628.624 | 0.388385 | 0.583 |
| Model 7 | -1261.118 | -18 | 2907.168 | 2646.235 | 0.000000 | 0.574 |
| Model 8 | -1261.118 | -27 | 2963.045 | 2664.235 | 0.000000 | 0.431 |
| Model 9 | -1261.118 | -36 | 3018.923 | 2682.235 | 0.000000 | 0.49 |
| Model 10 | -1261.118 | -45 | 3074.800 | 2700.235 | 0.000000 | 0.457 |


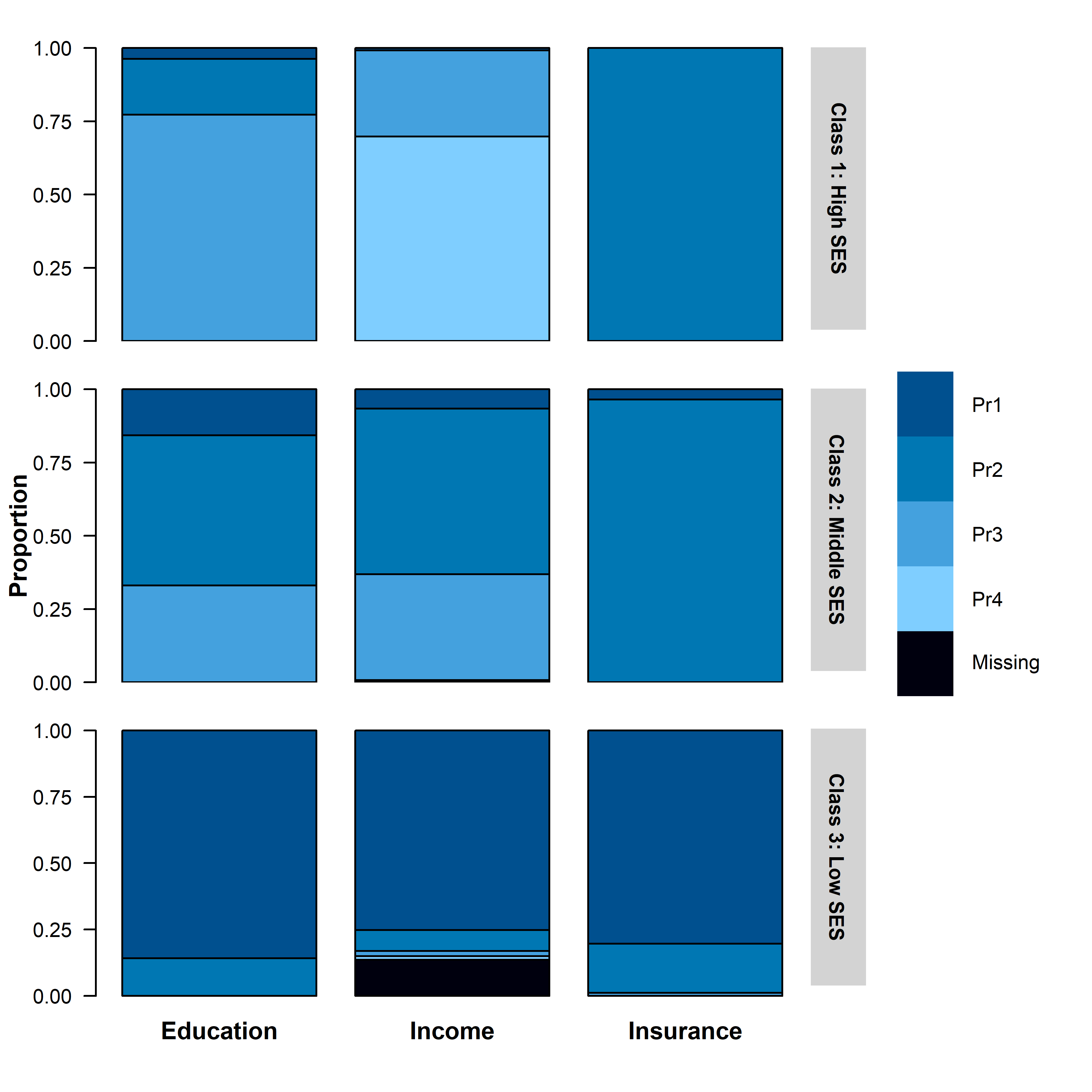


**Education:** Pr(1) pre-baccalaureate, Pr(2) baccalaureate, Pr(3) Post-baccalaureate

**Income:** Pr(1) lowest income, Pr(2) second quartile, Pr(3) third quartile, Pr(4) highest income, Pr(5) missing

**Insurance:** Pr(1): Medicaid/Medicare, Pr(2): Private, Pr(3) missing
