# Supplementary material for: Rurality, socioeconomic status, and psychosocial health outcomes during pregnancy
Source: BMC Pregnancy Childbirth. 2025 Dec 1;26:23. doi: 10.1186/s12884-025-08492-1 (PMC12777491; doi:10.1186/s12884-025-08492-1)
Supplement: Supplementary file 2 — Additional file 2. Unadjusted Differences in Psychosocial Outcomes by Rurality and Socioeconomic Categories Across Pregnancy. [file 12884_2025_8492_MOESM2_ESM.docx]

| **Additional File 2.** Unadjusted Differences in Psychosocial Outcomes by Rurality and Socioeconomic Categories Across Pregnancy. | | | | | | | | |  |
| --- | --- | --- | --- | --- | --- | --- | --- | --- | --- |
| Rurality | Socioeconomic Categories | n | CES-D | *P* | NVPQoL | *P* | PSS | *P* | |
| **Area Deprivation Index** | | | | | | | | |  |
| Urban | Least Disadvantage | 402 | 6.22 (0.34) | Ref | 86.10 (2.39) | Ref | 13.69 (0.51) | Ref | |
| Micropolitan Rural | Least Disadvantage | 10 | 4.48 (2.05) | 1.00 | 88.56 (14.50) | 1.00 | 14.64 (3.06) | 1.00 | |
| Small Town Rural | Least Disadvantage | 12 | 6.00 (1.99) | 1.00 | 77.92 (14.02) | 1.00 | 15.33 (2.98) | 1.00 | |
| Urban | Middle Disadvantage | 406 | 6.24 (0.34) | 1.00 | 84.16 (2.38) | 1.00 | 13.85 (0.51) | 1.00 | |
| Micropolitan Rural | Middle Disadvantage | 17 | 5.93 (1.55) | 1.00 | 79.71 (11.09) | 1.00 | 13.53 (2.31) | 1.00 | |
| Small Town Rural | Middle Disadvantage | 52 | 7.19 (0.95) | 0.99 | 91.68 (6.66) | 1.00 | 14.50 (1.41) | 1.00 | |
| Urban | Most Disadvantage | 253 | 7.67 (0.42) | 0.15 | 94.88 (2.94) | 0.34 | 15.49 (0.62) | 0.38 | |
| Micropolitan Rural | Most Disadvantage | 86 | 8.57 (0.72) | 0.08 | 97.73 (5.12) | 0.50 | 16.60 (1.07) | 0.26 | |
| Small Town Rural | Most Disadvantage | 127 | 8.27 (0.60) | 0.07 | 99.03 (4.20) | 0.16 | 15.96 (0.89) | 0.39 | |
| p-for-interaction |  |  |  | 0.75 |  | 0.88 |  | 0.98 | |
| **Individual Level Socioeconomic Status Latent Classes** | | | | | | | | |  |
| Urban | Class 1 (High SES) | 478 | 5.54 (0.30) | Ref | 81.93 (2.18) | Ref | 12.66 (0.46) | Ref | |
| Micropolitan Rural | Class 1 (High SES) | 28 | 5.40 (1.24) | 1.00 | 76.47 (8.88) | 1.00 | 13.53 (1.85) | 1.00 | |
| Small Town Rural | Class 1 (High SES) | 33 | 6.06 (1.17) | 1.00 | 92.24 (8.36) | 0.96 | 14.70 (1.75) | 0.97 | |
| Urban | Class 2 (Middle SES) | 472 | 7.13 (0.30) | 0.01 | 91.17 (2.18) | 0.07 | 15.43 (0.45) | <.001 | |
| Micropolitan Rural | Class 2 (Middle SES) | 48 | 8.20 (0.93) | 0.15 | 102.27 (6.70) | 0.09 | 15.95 (1.39) | 0.38 | |
| Small Town Rural | Class 2 (Middle SES) | 113 | 7.33 (0.62) | 0.20 | 92.54 (4.47) | 0.45 | 14.48 (0.93) | 0.71 | |
| Urban | Class 3 (Low SES) | 138 | 8.15 (0.54) | <.001 | 94.19 (3.87) | 0.13 | 15.21 (0.80) | 0.12 | |
| Micropolitan Rural | Class 3 (Low SES) | 37 | 8.90 (1.03) | 0.05 | 96.19 (7.62) | 0.68 | 17.57 (1.54) | 0.06 | |
| Small Town Rural | Class 3 (Low SES) | 46 | 10.17 (0.92) | <.001 | 105.95 (6.61) | 0.02 | 18.55 (1.37) | <.001 | |
| p-for-interaction |  |  |  | 0.60 |  | 0.32 |  | 0.20 | |

Abbreviations: CES-D = Center for Epidemiologic Studies Depression Scale, NVPQoL = Nausea and Vomiting Quality of Life Score, PSS = Perceived Stress Score.
