# Supplementary material for: Rurality, socioeconomic status, and psychosocial health outcomes during pregnancy
Source: BMC Pregnancy Childbirth. 2025 Dec 1;26:23. doi: 10.1186/s12884-025-08492-1 (PMC12777491; doi:10.1186/s12884-025-08492-1)
Supplement: Supplementary file 3 — Additional file 3. Adjusted Differences in Psychosocial Outcomes by Rurality and Socioeconomic Categories Across Pregnancy. [file 12884_2025_8492_MOESM3_ESM.docx]

| **Additional File 3.** Adjusted Differences in Psychosocial Outcomes by Rurality and Socioeconomic Categories Across Pregnancy. | | | | | | | | |
| --- | --- | --- | --- | --- | --- | --- | --- | --- |
| Rurality | Socioeconomic Category | n | CES-D | *P* | NVPQoL | *P* | PSS | *P* |
| **Area Deprivation Index** | | | | | | | | |
| Urban | Least Disadvantage | 402 | 6.92 (0.95) | Ref | 88.89 (6.70) | Ref | 13.46 (1.43) | Ref |
| Micropolitan Rural | Least Disadvantage | 10 | 4.83 (2.21) | 0.98 | 88.79 (15.62) | 1.00 | 14.23 (3.32) | 1.00 |
| Small Town Rural | Least Disadvantage | 12 | 6.97 (2.14) | 1.00 | 80.56 (15.15) | 1.00 | 15.29 (3.24) | 1.00 |
| Urban | Middle Disadvantage | 406 | 6.75 (0.92) | 1.00 | 85.84 (6.52) | 0.99 | 13.60 (1.39) | 1.00 |
| Micropolitan Rural | Middle Disadvantage | 17 | 6.25 (1.76) | 1.00 | 78.79 (12.59) | 0.99 | 12.67 (2.65) | 1.00 |
| Small Town Rural | Middle Disadvantage | 52 | 7.60 (1.29) | 1.00 | 92.64 (9.11) | 1.00 | 14.15 (1.94) | 1.00 |
| Urban | Most Disadvantage | 253 | 7.24 (0.95) | 1.00 | 92.19 (6.73) | 1.00 | 14.15 (1.43) | 1.00 |
| Micropolitan Rural | Most Disadvantage | 86 | 8.12 (1.13) | 0.89 | 94.93 (8.09) | 0.99 | 15.28 (1.71) | 0.88 |
| Small Town Rural | Most Disadvantage | 127 | 8.37 (1.07) | 0.49 | 98.36 (7.57) | 0.61 | 15.15 (1.61) | 0.81 |
| p-for-interaction |  |  |  | 0.69 |  | 0.84 |  | 0.95 |
| **Socioeconomic Latent Classes** | | | | | | | | |
| Urban | Class 1 (High SES) | 478 | 5.60 (0.97) | Ref | 81.62 (6.94) | Ref | 12.24 (1.43) | Ref |
| Micropolitan Rural | Class 1 (High SES) | 28 | 5.47 (1.53) | 1.00 | 75.63 (10.97) | 1.00 | 13.15 (2.30) | 1.00 |
| Small Town Rural | Class 1 (High SES) | 33 | 6.16 (1.47) | 1.00 | 91.13 (10.55) | 0.97 | 14.19 (2.21) | 0.98 |
| Urban | Class 2 (Middle SES) | 472 | 7.07 (0.92) | 0.02 | 89.01 (6.62) | 0.30 | 14.60 (1.39) | 0.01 |
| Micropolitan Rural | Class 2 (Middle SES) | 48 | 7.17 (1.34) | 0.85 | 95.74 (9.64) | 0.62 | 14.13 (2.01) | 0.95 |
| Small Town Rural | Class 2 (Middle SES) | 113 | 7.17 (1.10) | 0.38 | 87.97 (7.88) | 0.94 | 13.53 (1.65) | 0.95 |
| Urban | Class 3 (Low SES) | 138 | 6.82 (0.97) | 0.81 | 87.52 (6.98) | 0.98 | 12.72 (1.46) | 1.00 |
| Micropolitan Rural | Class 3 (Low SES) | 37 | 8.19 (1.33) | 0.43 | 91.68 (9.73) | 0.97 | 15.58 (2.00) | 0.64 |
| Small Town Rural | Class 3 (Low SES) | 46 | 9.62 (1.27) | <.001 | 105.74 (9.11) | 0.03 | 17.24 (1.91) | 0.04 |
| p-for-interaction |  |  |  | 0.33 |  | 0.17 |  | 0.05 |

CES-D = Center for Epidemiologic Studies Depression Scale, NVPQoL = Nausea and Vomiting Quality of Life Score, PSS = Perceived Stress Score. Adjusted for age, pre-pregnancy body mass index, marital status, race, and number of children living at home.
