# Supplementary material for: Rurality, socioeconomic status, and psychosocial health outcomes during pregnancy
Source: BMC Pregnancy Childbirth. 2025 Dec 1;26:23. doi: 10.1186/s12884-025-08492-1 (PMC12777491; doi:10.1186/s12884-025-08492-1)
Supplement: Supplementary file 4 — Additional file 4. Sensitivity Analyses (Excluding Pittsburgh site). [file 12884_2025_8492_MOESM4_ESM.docx]

**Additional File 4.** Sensitivity Analyses (Excluding Pittsburgh site)

| Sensitivity Analysis 1: Unadjusted Differences n Psychosocial Factors by Rurality and Socioeconomic Status Across Pregnancy. | | | | | | |
| --- | --- | --- | --- | --- | --- | --- |
| Group | CES-D | *P* | NVPQoL | *P* | PSS | *P* |
| **Rurality** | | | | | | |
| Urban (n=263) | 6.60 (0.25) | Reference | 88.43 (1.78) | Reference | 14.22 (0.36) | Reference |
| Micropolitan Rural (n=41) | 7.71 (0.64) | 0.24 | 93.54 (4.63) | 0.56 | 15.68 (0.92) | 0.30 |
| Small Town Rural (n=68) | 7.89 (0.49) | 0.05 | 95.98 (3.51) | 0.13 | 15.70 (0.71) | 0.15 |
| **Area Deprivation Index** | | | | | | |
| Least Disadvantage (n=106) | 6.17 (0.33) | Reference | 85.94 (2.32) | Reference | 13.76 (0.49) | Reference |
| Middle Disadvantage (n=133) | 6.33 (0.31) | 0.93 | 84.80 (2.19) | 0.93 | 13.91 (0.46) | 0.97 |
| Most Disadvantage (n=125) | 8.00 (0.31) | <.001 | 96.52 (2.17) | <.001 | 15.82 (0.46) | 0.01 |
| **Individual Socioeconomic Status Latent Class** | | | | | | |
| High SES (n=129) | 5.64 (0.33) | Reference | 83.16 (2.36) | Reference | 13.00 (0.48) | Reference |
| Middle SES (n=183) | 7.39 (0.30) | <.001 | 92.64 (2.17) | 0.01 | 15.26 (0.44) | <.001 |
| Low SES (n=60) | 8.93 (0.52) | <.001 | 101.66 (3.76) | <.001 | 16.91 (0.75) | <.001 |

CES-D = Center for Epidemiologic Studies Depression Scale, NVPQoL = Nausea and Vomiting Quality of Life Score, PSS = Perceived Stress Score.

| Sensitivity Analysis 2. Adjusted Associations of Rurality and Socioeconomic Status with Psychosocial Factors Across Pregnancy. | | | | | | |
| --- | --- | --- | --- | --- | --- | --- |
| Group | CES-D | *P* | NVPQoL | *P* | PSS | *P* |
| **Rurality** | | | | | | |
| Urban (n=263) | 7.14 (0.63) | Reference | 91.83 (4.46) | Reference | 15.33 (0.91) | Reference |
| Micropolitan Rural (n=41) | 7.40 (0.88) | 0.93 | 93.49 (6.31) | 0.94 | 15.93 (1.28) | 0.83 |
| Small Town Rural (n=68) | 8.08 (0.79) | 0.22 | 97.98 (5.59) | 0.27 | 16.26 (1.15) | 0.49 |
| **National Deprivation Index** | | | | | | |
| Least Disadvantage (n=106) | 6.85 (0.73) | Reference | 91.11 (5.12) | Reference | 14.87 (1.06) | Reference |
| Middle Disadvantage (n=133) | 6.80 (0.69) | 0.99 | 87.88 (4.86) | 0.64 | 14.90 (1.01) | 1.00 |
| Most Disadvantage (n=125) | 7.88 (0.68) | 0.16 | 98.34 (4.79) | 0.15 | 16.15 (0.99) | 0.26 |
| **Individual Socioeconomic Status Latent Class** | | | | | | |
| High SES (n=129) | 5.26 (0.78) | Reference | 80.96 (5.53) | Reference | 13.31 (1.14) | Reference |
| Middle SES (n=183) | 6.82 (0.72) | <.001 | 87.60 (5.12) | 0.10 | 15.06 (1.05) | 0.02 |
| Low SES (n=60) | 8.10 (0.70) | <.001 | 99.27 (5.00) | <.001 | 16.34 (1.03) | 0.02 |

Abbreviations: CES-D = Center for Epidemiologic Studies Depression Scale, NVPQoL = Nausea and Vomiting Quality of Life Score, PSS = Perceived Stress Score. Adjusted for age, pre-pregnancy BMI, marital status, race, and number of children living at home.

| Sensitivity Analysis 3. Interactions of Rurality by Socioeconomic Variables with Psychosocial Factors Across Pregnancy. | | | | | | | | |
| --- | --- | --- | --- | --- | --- | --- | --- | --- |
| Rurality | Socioeconomic Variable (n) |  | CES-D | *P* | NVPQoL | *P* | PSS | *P* |
| **Neighborhood Deprivation** | | | | | | | | |
| Urban | Least Disadvantage (281) |  | 6.37 (0.41) | Ref | 86.98 (2.89) | Ref | 13.68 (0.59) | Ref |
| Micropolitan Rural | Least Disadvantage (10) |  | 4.50 (2.07) | 0.99 | 88.66 (14.66) | 1.00 | 14.67 (2.99) | 1.00 |
| Small Town Rural | Least Disadvantage (12) |  | 6.00 (2.00) | 1.00 | 77.92 (14.18) | 1.00 | 15.33 (2.91) | 1.00 |
| Urban | Middle Disadvantage (312) |  | 6.40 (0.39) | 1.00 | 84.46 (2.75) | 1.00 | 14.01 (0.56) | 1.00 |
| Micropolitan Rural | Middle Disadvantage (17) |  | 5.95 (1.56) | 1.00 | 79.86 (11.21) | 1.00 | 13.55 (2.25) | 1.00 |
| Small Town Rural | Middle Disadvantage (52) |  | 7.19 (0.95) | 1.00 | 91.71 (6.73) | 1.00 | 14.50 (1.38) | 1.00 |
| Urban | Most Disadvantage (138) |  | 7.55 (0.58) | 0.76 | 98.87 (4.07) | 0.30 | 15.60 (0.83) | 0.63 |
| Micropolitan Rural | Most Disadvantage (80) |  | 8.52 (0.75) | 0.22 | 97.33 (5.36) | 0.75 | 16.31 (1.08) | 0.45 |
| Small Town Rural | Most Disadvantage (124) |  | 8.31 (0.61) | 0.17 | 99.06 (4.29) | 0.32 | 16.12 (0.88) | 0.34 |
| p-for-interaction |  |  |  | 0.73 |  | 0.85 |  | 0.99 |
| **Socioeconomic Latent Classes** | | | | | | | | |
| Urban | Class 1 (High SES) (354) |  | 5.62 (0.36) | Ref | 82.86 (2.56) | Ref | 12.80 (0.51) | Ref |
| Micropolitan Rural | Class 1 (High SES) (28) |  | 5.41 (1.25) | 1.00 | 76.51 (8.97) | 1.00 | 13.54 (1.80) | 1.00 |
| Small Town Rural | Class 1 (High SES) (33) |  | 6.06 (1.17) | 1.00 | 92.24 (8.44) | 0.98 | 14.70 (1.70) | 0.98 |
| Urban | Class 2 (Middle SES) (321) |  | 7.29 (0.37) | 0.03 | 91.17 (2.66) | 0.37 | 15.42 (0.54) | 0.01 |
| Micropolitan Rural | Class 2 (Middle SES) (48) |  | 8.21 (0.93) | 0.19 | 102.34 (6.76) | 0.15 | 15.97 (1.35) | 0.41 |
| Small Town Rural | Class 2 (Middle SES) (113) |  | 7.34 (0.63) | 0.30 | 92.57 (4.51) | 0.63 | 14.49 (0.91) | 0.79 |
| Urban | Class 3 (Low SES) (77) |  | 8.10 (0.74) | 0.07 | 101.54 (5.32) | 0.04 | 15.54 (1.07) | 0.34 |
| Micropolitan Rural | Class 3 (Low SES) (31) |  | 8.84 (1.12) | 0.14 | 94.90 (8.35) | 0.91 | 17.02 (1.62) | 0.24 |
| Small Town Rural | Class 3 (Low SES) (43) |  | 10.40 (0.95) | <.001 | 106.45 (6.87) | 0.04 | 19.12 (1.38) | <.001 |
| p-for-interaction |  |  |  | 0.52 |  | 0.32 |  | 0.23 |

| Sensitivity Analysis 4. Adjusted Associations of Rurality by Socioeconomic Variables with Psychosocial Factors Across Pregnancy. | | | | | | | |
| --- | --- | --- | --- | --- | --- | --- | --- |
| Rurality | Socioeconomic Variable (n) | CES-D | *P* | NVPQoL | *P* | PSS | *P* |
| **Neighborhood Deprivation** | | | | | | | |
| Urban | Least Disadvantage (281) | 6.99 (0.74) | Ref | 91.56 (5.21) | Ref | 13.68 (0.59) | Ref |
| Micropolitan Rural | Least Disadvantage (10) | 4.57 (2.14) | 0.96 | 90.81 (14.95) | 1.00 | 14.67 (2.99) | 1.00 |
| Small Town Rural | Least Disadvantage (12) | 6.90 (2.07) | 1.00 | 85.98 (14.48) | 1.00 | 15.33 (2.91) | 1.00 |
| Urban | Middle Disadvantage (312) | 6.75 (0.71) | 1.00 | 87.29 (4.98) | 0.97 | 14.01 (0.56) | 1.00 |
| Micropolitan Rural | Middle Disadvantage (17) | 6.13 (1.66) | 1.00 | 80.53 (11.73) | 0.99 | 13.55 (2.25) | 1.00 |
| Small Town Rural | Middle Disadvantage (52) | 7.51 (1.13) | 1.00 | 95.03 (7.93) | 1.00 | 14.50 (1.38) | 1.00 |
| Urban | Most Disadvantage (138) | 7.58 (0.79) | 1.00 | 98.20 (5.53) | 0.93 | 15.60 (0.83) | 0.97 |
| Micropolitan Rural | Most Disadvantage (80) | 7.98 (0.97) | 0.97 | 96.97 (6.88) | 0.99 | 16.31 (1.08) | 0.96 |
| Small Town Rural | Most Disadvantage (124) | 8.25 (0.87) | 0.76 | 100.14 (6.08) | 0.79 | 16.12 (0.88) | 0.91 |
| p-for-interaction |  |  | 0.69 |  | 0.84 |  | 0.95 |
| **Socioeconomic Latent Classes** | | | | | | | |
| Urban | Class 1 (High SES) (354) | 5.42 (0.80) | Ref | 81.54 (5.68) | Ref | 13.38 (1.17) | Ref |
| Micropolitan Rural | Class 1 (High SES) (28) | 5.23 (1.43) | 1.00 | 75.36 (10.12) | 1.00 | 14.14 (2.09) | 1.00 |
| Small Town Rural | Class 1 (High SES) (33) | 5.85 (1.36) | 1.00 | 91.25 (9.65) | 0.97 | 14.98 (1.99) | 0.99 |
| Urban | Class 2 (Middle SES) (321) | 7.02 (0.74) | 0.05 | 87.87 (5.29) | 0.73 | 15.60 (1.09) | 0.09 |
| Micropolitan Rural | Class 2 (Middle SES) (48) | 6.81 (1.22) | 0.93 | 94.93 (8.67) | 0.70 | 15.04 (1.78) | 0.98 |
| Small Town Rural | Class 2 (Middle SES) (113) | 6.90 (0.93) | 0.53 | 87.86 (6.62) | 0.95 | 14.45 (1.36) | 0.99 |
| Urban | Class 3 (Low SES) (77) | 7.40 (0.84) | 0.49 | 98.09 (5.96) | 0.26 | 15.06 (1.23) | 0.96 |
| Micropolitan Rural | Class 3 (Low SES) (31) | 8.17 (1.24) | 0.47 | 94.16 (9.08) | 0.92 | 16.73 (1.81) | 0.71 |
| Small Town Rural | Class 3 (Low SES) (43) | 9.59 (1.13) | 0.01 | 107.26 (8.02) | 0.03 | 18.69 (1.65) | 0.03 |
| p-for-interaction |  |  | 0.33 |  | 0.17 |  | 0.05 |
